# Signatures of selection in loci governing major colour patterns in *Heliconius *butterflies and related species

**DOI:** 10.1186/1471-2148-10-368

**Published:** 2010-11-29

**Authors:** Grace C Wu, Mathieu Joron, Chris D Jiggins

**Affiliations:** 1 Department of Zoology, University of Cambridge, Downing Street, Cambridge, CB2 3EJ, UK; 2 Museum National d'Histoire Naturelle, Paris, France

## Abstract

**Background:**

Protein-coding change is one possible genetic mechanism underlying the evolution of adaptive wing colour pattern variation in *Heliconius *butterflies. Here we determine whether 38 putative genes within two major *Heliconius *patterning loci, *HmYb *and *HmB*, show evidence of positive selection. Ratios of nonsynonymous to synonymous nucleotide changes (ω) were used to test for selection, as a means of identifying candidate genes within each locus that control wing pattern.

**Results:**

Preliminary analyses using 454 transcriptome and Bacterial Artificial Chromosome (BAC) sequences from three *Heliconius *species highlighted a cluster of genes within each region showing relatively higher rates of sequence evolution. Other genes within the region appear to be highly constrained, and no ω estimates exceeded one. Three genes from each locus with the highest average pairwise ω values were amplified from additional *Heliconius *species and races. Two selected genes, *fizzy-like *(*HmYb*) and *DALR *(*HmB*), were too divergent for amplification across species and were excluded from further analysis. Amongst the remaining genes, *HM00021 *and *Kinesin *possessed the highest background ω values within the *HmYb *and *HmB *loci, respectively. After accounting for recombination, these two genes both showed evidence of having codons with a signature of selection, although statistical support for this signal was not strong in any case.

**Conclusions:**

Tests of selection reveal a cluster of candidate genes in each locus, suggesting that weak directional selection may be occurring within a small region of each locus, but coding changes alone are unlikely to explain the full range of wing pattern diversity. These analyses pinpoint many of the same genes believed to be involved in the control of colour patterning in *Heliconius *that have been identified through other studies implementing different research methods.

## Background

Mechanisms by which biological information is encoded in living systems are incredibly diverse, and the resulting biological diversity is no less astounding. The relative contribution of different mechanisms to adaptive phenotypic variation has been the focus of many recent evolutionary and developmental studies and the subject of much debate [1-4]. One outstanding question is the relative preponderance of coding versus regulatory mutations in generating phenotypic change. Numerous studies have shown rapid evolution of protein coding regions of genes, for example in hominid lineages [5]. For other vertebrates, frequently cited examples of morphological variation controlled by amino acid changes include repeated involvement of *Melanocortin-1 receptor *(*Mc1r*) mutations in mice, birds, and reptiles in adaptive melanic pigmentation [6-8].

Increasingly, the importance of *cis*-regulatory elements (CREs) as the "creative" force in evolution has assumed center stage [9,3]. The theoretical basis for this hypothesis is that every gene possesses a modular arrangement of 5' CREs that control temporal and spatial patterns of expression, and that these regions are less subject to the negative pleiotropic effects constraining coding changes, allowing adaptive evolution to proceed at a higher rate [10]. Oft-cited examples of *cis*-regulatory change as the molecular basis for phenotypic variation come from *Drosophila*, notably studies of the genetic control of yellow pigmentation patterning in adult wings of *Drosophila biarmipes *[11]. Other well-examined morphological changes include the pelvic spine and armoured plate in sticklebacks and bristles in *Drosophila *larvae [12,13].

The *Heliconius *radiation presents an excellent opportunity to test these competing hypotheses in evolutionary genetics. These mimetic, aposematic butterflies are highly phenotypically varied and complex, yet patterns are under simple genetic control. Patterning variation exists on multiple levels, from inter-specific, through geographic variation within species to intra-population polymorphism, and can be attributed to strong selection for mimicry that also drives adaptive radiation [14,15]. Most importantly, the variation observed represents several cases of adaptive divergence and convergence, providing multiple sets of comparisons to test whether evolution recruits the same genetic pathways to produce similar phenotypic traits.

Joron et al. identified, through linkage mapping, a conserved locus responsible for control of wing patterning in three *Heliconius *species, suggesting that a conserved region controls pattern evolution across the genus [16]. In *Heliconius melpomene*, *HmYb *and *HmB *loci are two major regions known to control the hindwing bar element and the red forewing band, respectively. The corresponding loci in *Heliconius erato*, the co-mimic of *H. melpomene*, have been mapped to the same genomic regions as *HmYb *and *HmB*, suggesting the genetic mechanisms giving rise to shared phenotypes across co-mimicking races may be similar. Furthermore, these loci are characterised by multiple alleles responsible for many different phenotypes, such that we might expect the genes responsible to have undergone multiple rounds of strong positive selection. *HmYb *and *HmB *have recently been annotated, and between 20 and 24 genes have been predicted in each locus [17,18].

It is currently unknown whether coding or regulatory regions within these loci are primarily responsible for wing pattern diversity. In parallel studies we have been examining gene expression differences between races of *Heliconius melpomene*. To complement this approach, we here examine signatures of evolution in coding regions by estimating the ratio (ω) of nonsynonymous (*d_N_*) to synonymous changes (*d_S_*) in 20 and 18 putative patterning genes within the *HmYb *and *HmB *loci, respectively [19]. We first analysed BAC sequences, Expressed Sequence Tag (EST) transcripts, and 454 transcriptome sequences, most of which are available for three focal species (*Heliconius melpomene*, *Heliconius numata*, and *Heliconius erato*) and two races of *H. melpomene *(*H. m. cythera and H. m. malleti*). Three selected genes from each locus were then sequenced for the entire coding region in several lineages of *Heliconius *and a closely related genus when possible. The results identify clusters of three or four genes with elevated rates of evolution, but fail to provide strong statistical evidence for diversifying selection. Nonetheless, genes highlighted here as having high rates of protein coding evolution are good candidates for further investigation into their role in wing patterning, as has been recently found for the UV opsins and their role in *Heliconius *vision [20].

## Results

### Initial pairwise and lineage-wide estimates of *d_N _*and *d_S _*suggest candidate patterning genes

**Figure 1:**
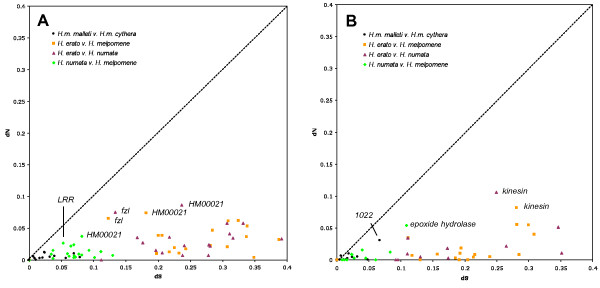
**Plot of *d_S _*v. *d_N _*values from pairwise PAML analyses for putative genes in the *HmYb *(A) and *HmB *(B) loci**. Pairwise tests between three polymorphic *Heliconius *species (*H. melpomene*, *H. numata*, *H. erato*) and two *H. melpomene *races (*H. m. cythera *and *H. m. malleti*) exhibiting higher than average *d_S _*and *d_N _*values, are indicated by putative gene names. While the majority of genes found in these color-patterning loci appear slow evolving and constrained, pairwise tests across species and races consistently highlighted the same outliers: hypothetical protein *HM00021 *(A), *Leucine-rich repeat (LRR) *(A), *fizzy-like *(*fzl*) (A), and *kinesin *(B). Other outliers, including *epoxide hydrolase *(B), show rapid rates of evolution in comparison to other genes for pairwise analyses only between *H. m. melpomene *and *H. numata*. The dashed line represents ω = 1.

Using available sequences of three *Heliconius *species and two races, we estimated the substitution rate for genes within the two patterning locus regions through 1) pairwise analyses with the YN00 program and 2) one-ratio branch model with CODEML in the PAML suite [21]. Since the only intra-specific comparison in these analyses is between the trans-Andean races, *H. m. malleti *and *H. m. cyrbia*, we consider recent recombination within sequences to be unlikely. Hence, we have explicitly accounted for recombination only in subsequent analyses with larger numbers of intra-specific comparisons. Generally low pairwise *d_N _*and *d_S _*values suggest that the majority of genes in the *HmYb *and *HmB *loci are slow evolving and highly constrained (Figure 1). Furthermore, no pairwise or background (one-ratio model) ω values of any gene in either locus exceeded one, which is the hallmark signature of positive selection. Conventionally, neutrality is inferred when ω = 1, purifying or negative selection when ω < 1, and positive or directional selection when ω > 1. However, the average ω value across sites in a gene is unlikely to be above 1 unless a large proportion of sites in the gene are under diversifying selection [22].

**Figure 2:**
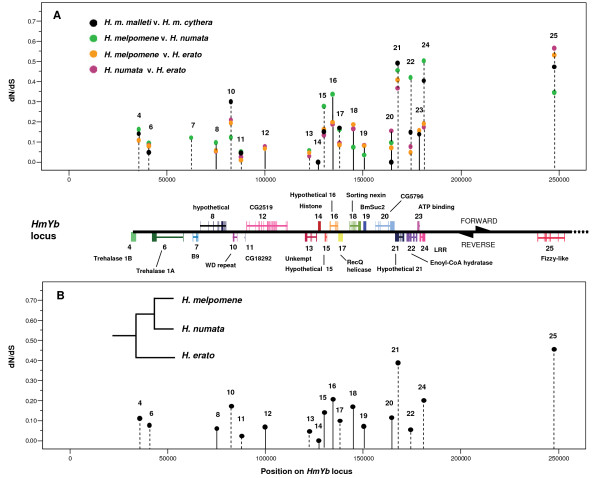
**Plot of initial pairwise (A) and lineage-based (B) ω values for putative genes in *HmYb***. Pairwise ω values for *HmYb*, the locus controlling the yellow hindwing bar, are plotted across the genomic region for *H. m. melpomene cythera *and *H. m. malleti *calculated using available 454 contigs, and the three focal *Heliconius *species, *H. erato*, *H. numata*, and *H. melpomene *using BAC sequences (A). The solid lines connecting data points indicate genes found on the forward strand, while the dotted lines indicate genes located on the reverse strand. Gaps in data reflect inadequate 454 contig coverage across races and species. Lineage-based analyses were used to estimate background ω values by averaging ratios over all sites and across all branches of the three focal polymorphic species, as illustrated in the inset tree topology (model = 0; NSsites = 0 in CODEML) (B). All plotted data are tabulated (Table 1).

Analysis of the 20 genes annotated within the *HmYb *region highlights three genes on the reverse strand and in close proximity to one another that show the highest average pairwise and background ω values: *HM00021*, *LRR *(*HM00024*), and *fizzy-like *(*fzl; HM00025*) (Table 1) [18]. Analysis of background ω values calculated across a phylogeny of available BAC sequences using the one-ratio model also pinpointed these same three genes, as well as *HM00016*, as having elevated values (Table 1; Figure 2). Of these genes, *fzl *yielded the highest lineage-wide ratio (ω = 0.456), which is also reflected in its highest average pairwise value (ω = 0.480; Table 1). All pairwise alignments of both *HM00021 *and *fzl *reveal more nonsynonymous changes than synonymous changes, while the number of nonsynonymous changes never exceeded synonymous changes for analyses of *HM00016*. Previous studies suggest ω > 0.5 to be the threshold at which adaptive evolution can be reasonably suspected [23]. Therefore, based on their ω estimates, genes *HM00021*, *LRR *and *fzl *were selected for further analyses using expanded datasets to explore possible adaptive evolution within the *Heliconius *radiation. These genes are clustered together along the *HmYb *BAC walk on the reverse strand (Figure 2).

**Table 1:** ω values estimated from transcriptome and BAC sequences of putative genes in HmYb

				*Pairwise*^4^																
G#^1^	Putative gene name	Total bps^2^	Lineage-based^3^	*H. m. malleti *v. *H. m. cythera*				*H. erato v. H. melpomene*				***H. erato *v**. *H. numata*				*H. numata *v. *H. melpomene*				*mean*
				
				bps	ω	N^5^	S^6^	Bps	ω	N^5^	S^6^	bps	ω	N^5^	S^6^	bps	ω	N^5^	S^6^	ω_s_
4	Trehalase 1B	1921	0.112	528	0.143	4	8	1740	0.110	48	147	1740	0.110	46	135	1740	0.165	21	35	0.132
6	Trehalase 1A	1776	0.076	1773	0.049	4	21	1773	0.081	31	108	1773	0.086	33	113	1773	0.096	9	29	0.078
7	B9 protein	570														567	0.122	6	13	0.122
8	hypothetical	2235	0.060					2232	0.057	18	167	2232	0.055	17	158	2232	0.099	9	46	0.070
10	WD repeat domain	1038	0.172	1035	0.302	2	2	1035	0.195	30	52	1035	0.211	27	45	1035	0.124	7	22	0.208
11	CG18292	351	0.024	351	0.047	1	6	351	0.011	1	30	351	0.027	2	24	351	0.053	1	5	0.035
12	CG2519	3129	0.071					3129	0.068	48	264	3129	0.080	51	240	3129	0.069	11	64	0.072
13	Unkempt	1812	0.047					1812	0.048	15	103	1812	0.031	10	103	1812	0.059	10	64	0.046
14	Histone H3	408	0.000	408	0	0	0	408	0	0	0	408	0	0	11	408	0	0	0	0
15	hypothetical	408	0.141	408	0.152	1	2	408	0.163	15	24	408	0.135	14	23	408	0.279	3	3	0.182
16	hypothetical	819	**0.207**					819	0.199	38	62	819	0.190	36	62	819	0.339	13	14	**0.243**
17	RecQ helicase	1896	0.100	912	0.171	2	3	1896	0.086	29	87	1896	0.095	35	91	1896	0.163	15	20	0.129
18	sorting nexin	1056	0.168					1053	0.188	28	67	1053	0.167	27	69	1053	0.076	2	12	0.144
19	BmSuc2	1491	0.072					1491	0.083	35	152	1491	0.086	37	153	1491	0.036	4	40	0.068
20	CG5796	621	0.115	681	0	0	2	621	0.073	13	61	621	0.157	13	25	621	0.098	4	10	0.082
21	hypothetical	945	**0.388**	945	0.492	9	4	945	0.409	86	54	945	0.369	69	36	945	0.457	29	14	**0.432**
22	Enoyl-CoA hydratase	891	0.055	891	0.149	4	8	891	0.049	7	43	891	0.077	10	45	891	0.421	11	8	0.174
23	ATP Binding	288		288	0.139	1	2	288	0.158	12	18									0.149
24	LRR	1419	**0.200**	1419	0.4058	3	2	1419	0.192	72	82	1419	0.176	68	86	1419	0.504	30	16	**0.319**
25	Fizzy-like	1179	**0.456**	1179	0.474	9	7	1179	0.533	57	38	1179	0.567	67	40	1179	0.347	21	20	**0.480**

	**Average**		0.191		0.168				0.142				0.137				0.231			0.167

^1^G#: Unique gene number used in original annotation and in the BAC walk in Fig. 2. Here truncated from original format "HM000..."

^2^Total bps: total length, in basepairs, of the annotated gene.

^3^Lineage-based: *Heliconius *lineage-based background ω estimates ('branch model = 0' in CODEML) using BAC sequences of three species (*H. melpomene*, *H. erato*, *H. numata*). Entire length of each gene was used for this analysis.

^4^Pairwise: Results of pairwise analyses (YN00 in PAML) between two *H. melpomene *races and between three *Heliconius *species are presented. The number of changes between two sequences for each gene was calculated by taking the product of the substitution rate per site (*d_N _*and *d_S_*) and the number of sites (N or S bases). Bps indicate the length of the nucleotide sequences used for each pairwise analysis.

^5^N: N*d_N _or the number of nonsynonymous changes

^6^S: S*d_S _or the number of synonymous changes

Bold formatting indicates genes with ω > 0.19 (average background ω value from lineage wide analyses). ω values were not estimated for base changes of three or fewer.

**Figure 3:**
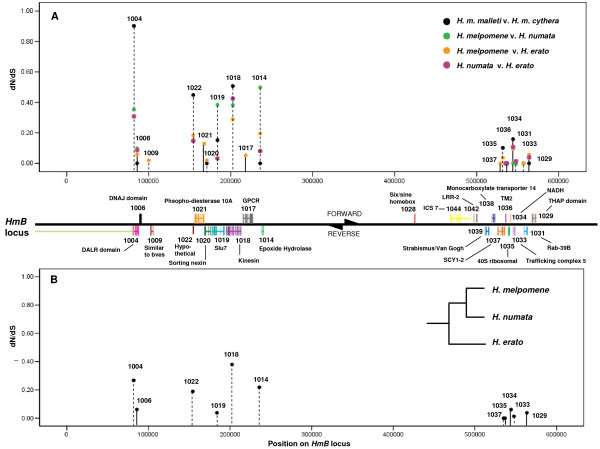
**Plot of initial pairwise (A) and lineage-based (B) ω values for putative genes in *HmB***. The same PAML analyses used to generate Fig. 2 were performed on 454 contigs and BAC sequences of genes within the *HmB *locus, which controls presence of the red band on the forewing. Genes with higher than average ω values across many pair-wise (A) and lineage-wide (B) analyses include *DALR *(*HM01004*), hypothetical protein (*HM01022*), and *kinesin *(*HM01018*). The solid lines connecting data points indicate genes found on the forward strand, while the dotted lines indicate genes located on the reverse strand. The background ω could be estimated for only a limited number of genes since there is no *HmB *BAC sequence for *H. numata*. Background ω values were calculated using available 454 contigs of *H. numata*.

In the *HmB *locus no pairwise, lineage-wide, or specific branch model tests estimated ω values that exceeded one, though *DALR *(*HM01004*), *kinesin *(*HM01018*), *HM01022*, and *epoxide hydrolase *(*HM01014*), exhibited pairwise ω values approaching 0.5 and high background ω values (Table 2; Figure 3). However, *epoxide hydrolase *(background ω = 0.219), has been slow evolving within *H. melpomene*, with no nonsynonymous changes and only three synonymous changes between *H. m. malleti *and *H. m. cythera*, which is insufficient to accurately estimate ω (Table 2). *HM01022 *and *DALR *show stronger signatures of selection in more recently diverged taxa, with the number of nonsynonymous changes exceeding synonymous changes between the two *H. melpomene *races (Figure 3ATable 2). Similar to the trend observed in *HmYb*, genes possessing high ω values in *HmB *are found on the same strand and are located in close proximity to each other along the locus, albeit interspersed with genes showing lower ω values (Figure 3).

**Table 2:** Preliminary ω estimates using available 454 and BAC sequences of putative genes in *HmB*

				Pairwise																
G#^1^	Putative gene name	Total bps	Lineage-wide	*H. m. malleti * v. *H. m. cythera*				*H. erato *v. *H. melpomene*				***H. erato *v**. *H. numata*				***H. numata *v**. *H. melpomene*				Mean
				
				bps	ω_s_	N	S	bps	ω	N	S	bps	ω	N	S	bps	ω	N	S	ω
1004	*DALR*	1242	**0.268**	1242	0.902	6	2	1242	0.309	32	34	1242	0.308	32	35	1242	0.353	8	6	**0.468**
1006	DNAJ binding domain	1014	0.062	1014	0	0	3	1014	0.058	5	32	1014	0.087	7	31	1014	0.098	2	7	0.061
1009	similar to bves	285						285	0.020	1	18									0.020
1022	hypothetical protein	441	**0.189**	441	0.448	11	6	411	0.183	17	30	411	0.148	16	33	411	0.144	4	6	**0.231**
1021	Phospho-diesterase	1947						1947	0.130	60	139									0.130
1020	sorting nexin	492		492	0	0	0	492	0.019	2	26									0.095
1019	slu7	1719	0.039	1719	0.153	6	16	1353	0.029	8	108	1353	0.031	11	120	1353	0.384	15	14	0.149
1018	kinesin-like	3735	**0.393**	2355	0.508	18	9	3735	0.290	242	214	1587	0.425	139	68	1587	0.381	11	7	**0.401**
1017	G-protein receptor	4623						4623	0.052	34	235									0.052
1014	epoxide hydrolase	252	**0.219**	252	0	0	3	252	0.196	18	31	252	0.081	4	16	252	0.499	10	7	**0.194**
1028	six/sine homeobox	753						753	0	0	124									0
1037	SCY1-like	2490		1515	0.101	2	6	2490	0.037	15	134									0.069
1036	TM2 domain	528	0	528	0	0	2	528	0	0	16	528	0	0	13	528	0	0	7	0
1035	40 S ribosomal protein	453	0	453	0	0	0	453	0	0	0	453	0	0	13	453	0	0	2	0
1034	NADH	348	0.062	348	0.159	1	2	348	0.095	5	14	348	0.107	5	13	348	0	0	1	0.090
1033	trafficking protein complex 5	558	0.013	558	0	0	0	558	0.013	1	26	558	0.014	1	24	558	0	0	1	0.007
1031	Ras-related	666		279	0	0	0	666	0	0	29									0
1029	THAP domain	1014	0.039	1014	0	0	1	1014	0.055	7	30	1014	0.038	4	28	1014	0.055	2	9	0.037

	Average		0.089		0.162				0.082				0.113				0.174			0.111

^1^G#: Unique gene number used in original annotation and in the BAC walk in Fig. 2. Here truncated from original format "HM0..."

Bold formatting indicates ω > 0.18. Other details are as indicated for Table 1.

The *kinesin *gene, in addition to yielding the highest background ω value (ω = 0.393; Table 2; Figure 3B) and second highest average pairwise ω value (ω = 0.401; Table 2), is the only gene to possess more nonsynonymous changes than synonymous changes across all pairwise analyses (Table 2). It is important to note that *kinesin *is an unusually large gene, and most pairwise analyses only encompassed 2 kilobases at the 3' end, since 454 contigs of *H. m. malleti*, *H. m. cythera*, and *H. numata *only span this region (Table 2). SMART (Simple Modular Architecture Research Tool) functional domain searches located a conserved *kinesin *motor domain at the 5' end, with the 3' end likely conferring protein binding specificity (for discussion of *kinesin *gene structure, see [17]) [24]. Thus, our analyses do at least include the region in which functionally significant changes are most likely to occur. Due to an abundance of insertions and deletions in exon 13 between *H. melpomene *and *H. erato*, the amino acid sequences were aligned with ClustalW and only nucleotides of aligning amino acids were used in tests of selection (Additional file 1).

### Tests of selection across closely and distantly related Heliconiini in selected candidate genes

Genes singled out from preliminary analyses were further examined by amplifying sequences across other Heliconiini lineages. In addition to *HM00021 *and *LRR*, *trehalase 1A *(*HM00006*) was included in further analyses since its gene expression was found to be associated with wing patterning (R. Reed, University of California, Irvine, personal correspondence; N.J. Nadeau et al., unpublished data). Unfortunately, we were unable to amplify *fzl *sequences from other races and species, precluding further investigation. For the *HmB *locus, *Mad *(*HM01000*) was included in these expanded analyses due to its potentially important role as a mitotic checkpoint protein, suggesting potential interactions with *fzl *in the *HmYb *locus [25]. *Mad *had been excluded from preliminary analyses due to a lack of 454 raw reads for the gene, and we were unable to amplify *DALR *from other races and species.

Since genealogical topologies may differ from published *Heliconius *phylogenies, trees for each of the candidate genes were reconstructed by maximum likelihood using the dnadist program in Phylip 3.69. Results show that all six candidate genes produce an overall topology similar to a recent *Heliconius *phylogeny [26]. The only discrepancy was placement of *H. charithonia*, a monomorphic species, within *H. erato *for *Mad*. However, because the bootstrap value for the branch grouping *H. charithonia *with *H. erato cyrbia *was low (34%), the published phylogeny was used for analysis.

### *HmYb *candidate genes show signatures of accelerated evolution

**Figure 4:**
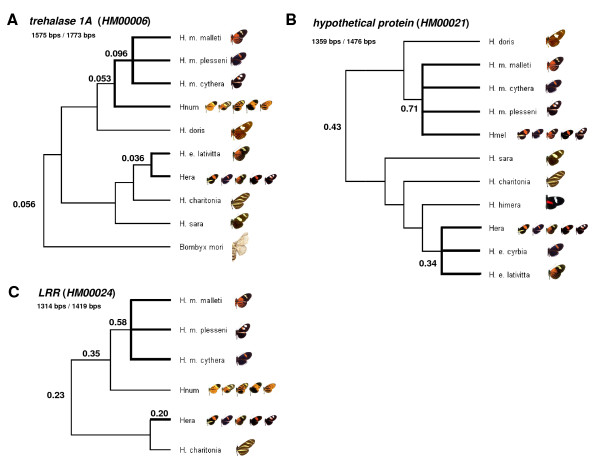
**Estimated ω values of *HmYb *candidate genes across various Heliconiini lineages**. Background and lineage-specific ω values were calculated for gene sequences amplified from various *Heliconius *species and races. Background ω values, calculated using the *one-ratio *model in CODEML, are indicated at the base of each unrooted tree. Bolded branches indicate the major polymorphic clades for wing pattern (*H. melpomene*, *H. erato*, *H. numata*). The topology of each tree was generated using amplified gene sequences by maximum likelihood. The *multiple ratios *model was used to estimate lineage specific ω values for the focal polymorphic species, *H. melpomene*, *H. numata*, and *H. erato *for three selected genes in the *HmYb *region: *trehalase 1A *(A), *HM00021 *(B), *LRR *(C). In addition to amplified sequences, BAC sequences are included and indicated by shorthand: Hera (*H. erato*), Hnum (*H. numata*), Hmel (*H. melpomene*). The number of coding nucleotide bases successfully sequenced and used in PAML analyses is shown over the gene total.

The estimated background ω value for *HM00021 *across 11 *Heliconius *taxa (ω = 0.43; Figure 4B) was comparable to the preliminary estimate that included only the three focal *Heliconius *taxa (ω = 0.39; Table 1). Nucleotide sequence alignments of exon 5 revealed several indels, some across many lineages, others specific between two species (Additional file 2A, B). However, indels were removed within reading frame from sequences used in all preliminary and expanded PAML analyses. Most notably, there is a 12 nucleotide insertion in *H. charithonia*, *H. doris*, *H. numata*, and *H. m. malleti *relative to the *H. melpomene *and *H. erato *BAC sequences (Additional file 2A). The amino acid alignments of exon 5 suggest that *H. doris *is an outgroup to the main *Heliconius *clades at this gene (percent similarity between *H. melpomene *and *H. doris*, 70%; and between *H. melpomene *and *H. erato*, 81%; Additional file 2B).

Since phylogeny-based methods of detecting positive selection do not take into consideration the effects of recombination on sequence evolution, gene alignments were analyzed using GARD (Genetic Algorithms for Recombination Detection) to estimate recombining breakage points [27,28]. Trees generated from the six recombinant fragments for *HM00021 *were then used in FEL (Fixed Effects Likelihood) tests for positive selection (Table 3). Following previous analyses, we take p < 0.1 as indicative of positive selection [28]. FEL analyses of GARD trees and the one neighbour joining tree found one codon with a signature of positive selection at 0.1 > p > 0.01 (Table 3). The inclusion or exclusion of race sequences did not affect detection of sites under directional selection (Table 3).

**Table 3:** Results of GARD and FEL analyses

	Races included^1^					Races excluded^2^			
**Gene**	**BP^3^**	**ΔAIC_C_^4^**	**KH p ≤ 0.05^5^**	**FEL (GARD)^6^**	**FEL (NJ)^7^**	**BP^3^**	**ΔAIC_C_^4^**	**KH p ≤ 0.05^5^**	**FEL (GARD) ^6^**

*Trehalase*	2	34.25	0	0	0	5	45.5	0	0
*HM00021*	5	265.8	2	403 (p = 0.06)	403 (p = 0.06)	7	199.13	0	403 (p = 0.04)
*HM00024*	2	129.72	0	0	0	2	120.03	1	0
*MAD/MAX*	3	14.53	0	0	0	2	6.03	0	0
*Kinesin*	8	596.42	2	154 (p = 0.09)	71 (p = 0.06); 154 (p = 0.08)	9	205.46	0	0
*HM01022*	0	N/A	N/A	N/A	0	2	0.2	0	0

^1^Races included: HyPhy analyses were performed using interspecific and intraspecific sequences.

^2^Races excluded: HyPhy analyses were performed using only interspecific sequences.

^3^BP: Number of recombinant breakage points predicted by GARD.

^4^ΔAIC_C_: The difference in AIC_C _score of the best GARD model that allows changing tree topologies for predicted partitions and the AIC_C _score for the null model in which one tree is inferred.

^5^KH p ≤ 0.05: Number of GARD predicted breakpoints that show significant topological incongruence by the Kuchibhatla and Hart (KH) test at p ≤ 0.05.

^6^FEL (GARD): Fixed Effects Likelihood (FEL) analysis detected codons showing signatures of positive selection at p ≤ 0.1 using topologies inferred by GARD recombinant partitions.

^7^FEL (NJ): Fixed Effects Likelihood (FEL) analysis detected codons showing signatures of positive selection (ω >1) at p ≤ 0.1 using a single neighbour joining (NJ) tree.

Due to the difficulty of amplifying the entire *LRR *gene (*HM00024*) in *H. erato *and other species in the pupal mating clade, analyses included only *H. charithonia *(a monomorphic species closely related to *H. erato*), three races of *H. melpomene *(*malleti*, *cythera*, *plesseni*), and BAC sequences of *H. erato *and *H. numata*. The background ω (0.23; Figure 4) across this topology is similar to that of the preliminary background analysis (ω = 0.20; Table 2). Three breakage points were detected by GARD analyses, and FEL tests of selection using these recombinant partitions found no sites undergoing positive selection (Table 3). Phylogeny-wide analyses reveal a low background ω value for *trehalase 1A *(ω = 0.074), and FEL tests of selection provided no evidence for selection.

### HmB genes

Due to difficulty in amplifying the entire length of *kinesin *from many species, analysis of *kinesin *was limited to exon 13 (1748 bps), the largest exon and also the region that includes the predicted "binding domain" of the protein. Because exon 13 contained an abundance of non-frame-shifting indels, amino acid sequences for all races were first aligned with ClustalW and nucleotide sequences of only overlapping amino acid positions were included in the PAML, GARD, and FEL analyses. The *H. doris kinesin *sequence is again highly divergent from the other *Heliconius *species sampled (Additional file 1).

**Figure 5:**
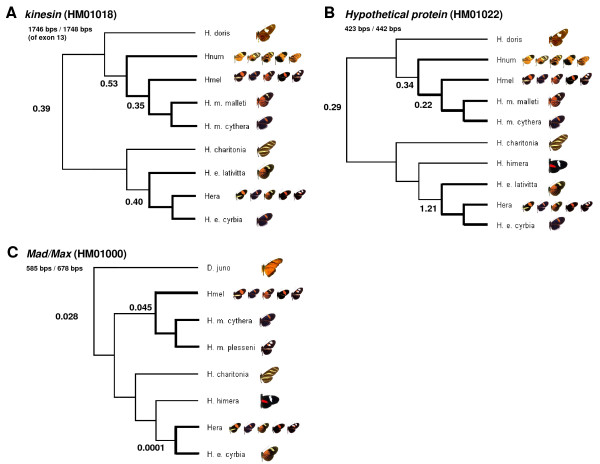
**Estimated ω values of *HmB *candidate genes across various Heliconiini lineages**. Background ω values are indicated at the base of each unrooted tree, calculated using the *one-ratio *model in PAML. The multiple ratios model was used to estimate lineage specific ω values for *H. melpomene*, *H. numata*, and *H. erato*, the three highly polymorphic species, of three genes in the *HmB *region: *MAD *(A), *kinesin *(B), and *HM01022 *(C). Other details regarding analyses and trees are as stated for Fig. 4.

*Kinesin *ω values are the highest estimated across all genes (Figure 5). Background, *H. melpomene *branch (*malleti*, *cythera*, *H. melpomene *BAC), and *H. erato *branch (*lativitta*, *cyrbia*, and *H. erato *BAC) ω values are similar: 0.39, 0.35, and 0.40, respectively (Figure 5B). GARD analyses generated eight predicted breakage points, two of which showed statistically significant topological incongruence at p = 0.05 (Table 3). One codon was found to show evidence for positive selection when accounting for recombination, while two codons were identified when a single NJ tree was used for analysis (Table 3).

*HM01022 *showed no evidence for recombination (Table 3), but also did not show any support for positive selection. Estimated *d_N_/d_S _*ratios for *Mad *are low, suggesting that the gene is also highly constrained (Figure 5A). Background ω was 0.034 and branch ω values for *H. melpomene *(*cythera *and *plesseni *races only) and *H. erato *were 0.071 and 0.0001, respectively (Figure 5). Accounting for recombination did not alter findings that there is no evidence for positive selection in *HM01022 *or *Mad *(Table 3).

## Discussion

The genetic basis of adaptation in natural populations is an area of considerable current interest in evolutionary biology. One major area of debate is the relative importance of protein-coding versus regulatory change in morphological adaptation. The *Heliconius *butterflies show both divergence and convergence in wing pattern, making them an interesting system to test these opposing hypotheses. Here, we explore protein-coding change across two genomic regions controlling wing pattern variation across multiple species of *Heliconius*. The role of these regions in wing pattern evolution across multiple species of *Heliconius *makes this a promising system to search for signatures of rapid evolution. However, our results provide little convincing evidence for accelerated protein-coding evolution by conventional interpretation. Although statistical support is mixed and ω values are never significantly greater than 1, a handful of genes do show evidence for adaptive evolution based on conservative estimates of ω ≈ 0.5. Nonetheless, both genomic regions studied provide some evidence for a clustering of genes with elevated ω, suggesting the influence of selection on the region. Furthermore, there is intriguing concordance between the genes highlighted here as having signatures of rapid evolution, and those already identified by gene expression and population genetic surveys.

### Fast evolving genes are clustered in candidate regions

Of the 20 genes analyzed for signatures of positive selection in the *HmYb *locus, only three genes, *HM00021*, *LRR*, and *fizzy-like*, consistently showed accelerated rates of evolution. And of the 18 examined genes in the *HmB *locus, *DALR*, *HM01022*, and *kinesin *yielded the highest ω values from several pairwise and lineage-wide tests of selection. It is striking that these candidate genes group together along the BAC walk: at the 5' end of the *HmB *locus and at the 3' of the *HmYb *locus, suggesting that in both cases a specific region in each locus may have undergone selection. While *HM00022 *is also found proximally to these genes along the same strand in *HmYb*, its ω values are relatively much lower. Since *HM00022 *codes for a protein that serves a known important function (Enoyl-CoA Hydratase), it may have experienced greater selective pressure to be functionally constrained compared to other genes discussed here [29]. These high ω values may indicate selection on the protein sequence of several functionally important genes, but perhaps more likely also indicate fixation of "nearly neutral mutations" rather than positive selection [30]. Genetic hitch-hiking due to repeated selective sweeps, perhaps on neighbouring *cis*-regulatory regions or untranslated regions (UTRs), could have locally reduced the effective population size, leading to fixation of nearly neutral coding changes [30]. Thus, repeated selective sweeps in close proximity to these genes would be expected to give a pattern of elevated ω values as observed. Regions of low recombination also experience a higher rate of nonsynonymous polymorphism and greater rate of accumulation of nonsynonymous substitutions over time that better reflect effective population size effects rather than positive selection [30-33]. Higher ω values could also therefore result from lower rates of recombination in these gene regions.

### The problem of polymorphism

The general assumption of the analyses performed in this present study is that differences between sequences are fixed differences between the populations studied. Of course, this will not be true in many cases, and in particular, closely related races will share polymorphisms. Thus, what tests of positive selection assume to be substitutions reflecting fixed changes may actually be "pre-fixations" [34]. Taking this into consideration, Kryazhimskiy and Plotkin suggest that ω values below one may in fact still be a signature of strong *or *weak positive selection, and this is supported by studies finding statistical evidence for adaptive evolution when ω > 0.5 with limited sequence comparisons [23,34]. In some cases single nucleotide polymorphisms were identified in the sequences between *H. m. cythera *and *H. m. malleti*, in which case the shared base was chosen for sequence analyses (see *Methods *section). This may lead to either artificially high or low ω estimates depending on whether polymorphic sites account for silent or non-silent variation. *HM00021 *(*HmYb*) was the only gene for which high polymorphism observed in 454 sequence reads was of concern. The potential consequences of "majority-rule" or "minority-rule" selection of polymorphic sites was addressed by running analyses on many possible combinations of polymorphic sites, with no strong effect on estimated ω values.

### Candidates in the *HmYb *locus

The ranges of many *Heliconius *species are broken up into divergent wing pattern races separated by narrow hybrid zones. Analysis of population differentiation for the *HmYb *locus between such wing pattern races showed a striking peak of population differentiation around the *LRR *gene in several pairwise comparisons [17]. Here, we have also highlighted this region, with *HM00021*, *LRR *(*HM00024*), and *fizzy-like *(*HM00025*) being relatively fast evolving.

In the *HmYb *locus, the only gene with tentative evidence for positive selection was *HM00021*. Its analyzed data set is more robust as it includes broad representation of closely related species outside of the *H. melpomene *and *H. erato *branches. Yet, estimated rates of synonymous and nonsynonymous substitutions are likely a conservative estimate of the true degree of sequence divergence, since alignments revealed several indels in the fifth and last exon of *HM00021 *(Additional file 2). While the majority of indels exist between species, a conspicuous 12 base-pair insertion in 454 contigs of *H. m. malleti *was found in exon 5 relative to the *H. melpomene *and *H. erato *BAC sequences (Additional file 2A). The method by which ω is estimated fails to capture and quantify the effects of insertions and deletions as part and parcel of "protein-coding" mutations, as they are theoretically equally capable as point mutations in affecting phenotype. While detailed examination of the distribution of synonymous and nonsynonmyous substitution sites in *HM00021 *revealed no discernable pattern, indels are notably only found in exon 5. However, little more can be inferred about *HM00021*'s candidacy as a hindwing pattern switch gene since it shows no homology to any known gene and does not contain recognisable protein motifs. Amplified sequences that contain indels maintain an open reading frame until the predicted 3' stop codon, suggesting that *HM00021 *is functionally active.

Though we were unable to successfully amplify sequences of *fizzy-like *from related species and races, initial ω estimates for *fzl *are amongst the highest, identifying it as a promising candidate gene. Furthermore, the gene shows highly complex patterns of alternative splicing between races of *H. melpomene *that correlate with hindwing phenotype (N.J. Nadeau et al., unpublished data). Strong 5' UTR divergence in *fzl *has been observed between races of *H. melpomene*, perhaps hinting at 5' UTR involvement in regulation of alternative splicing (N.J. Nadeau et al., unpublished data). While a great diversity of regulatory mechanisms for alternative splicing exist, including usage of regulatory modules that have been found in exons and introns, recent studies point to alternative 5' UTRs as important mechanisms in differential, complex, and highly targeted gene regulation [35,36]. Such findings, in conjunction with results in this present study, suggest that this gene may be a target for selection, either within the coding region, or on neighbouring non-coding regions that regulate splicing in *fzl*.

### Candidates in the *HmB *locus

In population genetic surveys between two Peruvian races of *H. melpomene*, a region of the *HmB *locus surrounding the *kinesin *gene was shown to be highly differentiated [17]. Even more compelling, gene expression studies showed parallel expression changes between races of *H. melpomene *and *H. erato *in exon 13 of the *kinesin *gene and *in-situ *hybridization experiments suggest consistent spatial and temporal patterns of gene expression on the forewing [37]. At *kinesin*, rates of evolution are relatively high along both the *H. erato *and *H. melpomene *branches, though ω values do not significantly differ from each other or from the background ratio (Figure 5B). Alignments of *kinesin *sequences amplified from many species and races revealed not only an abundance of SNPs, but also a profusion of indels that maintained open reading frames, suggesting that rapid sequence evolution had not simply resulted in non-functional proteins. Between the BAC sequences of *H. erato *and *H. melpomene *across exon 13, there are at least nine indels ranging in size from one to eight amino acids (Additional file 1A). Surprisingly, indel patterns do not agree with overall sequence divergence patterns in that *H. melpomene *and *H. charithonia*, species belonging to each of the two main clades, appear to share many of the amino acid indels (Additional file 1A). Together, the evidence supports *kinesin*'s strong candidacy as a potential *HmB *switch gene.

Not surprisingly, these very genes of interest identified in preliminary BAC analyses as the most "fast evolving," such as *fzl*, *DALR*, and *kinesin*, were difficult to amplify across species for further phylogeny-based analyses. Thus, in some ways our methods are biased against the most interesting genes. Fortunately however, analyses using BAC sequences available for three *Heliconius *species include all genes in the region, so are not biased in this way.

## Conclusions

The present study did not find strong support for protein-coding changes, at least those detectable through codon-based analyses, to be responsible for the control of major wing pattern elements in *Heliconius*. However, these analyses have been valuable in highlighting regions within each locus that show some signature of selection. Such patterns hint at the possibility of selection primarily acting on non-coding or regulatory regions found between or proximal to those genes, which have reduced effective population sizes in surrounding genomic regions. Interestingly, the identification of these gene regions corroborates the findings of related research efforts.

Over recent years, a paradigm has emerged for the origin of novel morphological adaptations that involves co-option of genes with conserved functions through novel regulatory interactions. However, this paradigm has arisen from studies using methods that are inherently biased towards discovering roles for conserved genes, such as the use of cross-staining antibodies. Here, we have found higher rates of sequence evolution in genes showing little or no homology to known proteins. The strong candidate gene for the yellow band (*HmYb*), *fizzy-like *(*HM00025*) shares at most only 26% amino-acid identity with other members of the *fizzy*-family. Findings from this present study raise the suspicion that some genes involved in wing colour patterning of *Heliconius *may be evolving so rapidly that homology cannot be easily established.

## Methods

### Sequence data acquisition for preliminary analyses

Protein-coding sequences of all predicted genes within the *HmYb *and *HmB *loci were used to search the NCBI Lepidoptera nucleotide and EST databases using tBLASTn to acquire *Bicyclus*, *Papilio*, *Bombyx*, *Spodoptera *and any related Lepidoptera nucleotide sequences. We searched consensus EST contigs available on Butterflybase http://butterflybase.ice.mpg.de/ and 454 transcriptome sequences using local BLAST, which were available for the following species/races: *Heliconius melpomene cythera *(short read archive accession number SRX005618), *Heliconius melpomene malleti *(SRX005617), *Heliconius numata *population 1 (Tarapoto, Peru, Huallaga biogeographic region; SRX014029), *Heliconius numata *population 2 (Yurimaguas, Peru, Ucayali biogeographic region; SRX014035), and *Heliconius erato *[18].

### Preliminary pairwise and lineage-wide evolutionary analyses

Using CodonCode alignment software, 454 consensus sequences and raw reads of each species or race for each gene were aligned against the BAC sequence to ensure that the transcripts were from the genes of interest. After eliminating misaligning transcripts, sequences were assembled into one contig per taxon per gene.

Contigs were truncated to give a core region of maximum overlap between all species and compiled into input files formatted for analysis using PAML (Phylogenetic Analysis by Maximum Likelihood; Ziheng Yang, Department of Biology, University College London). Input files were analyzed with the YN00 program using the method of Yang and Nielsen [38] to estimate pairwise rates of non-synonymous (*d_N_*) and synonymous substitutions (*d_S_*) between taxa (run mode = -2). If BAC sequences were available for all three *Heliconius *species of interest (*H. melpomene*, *H. numata*, and *H. erato*), the *one-ratio *model was applied using the CODEML program in PAML, with fixed rates (model = 0, NSsites = 0), to calculate a single background *d_N_*/*d_S _*(ω) for each tree per gene. Occasionally, complete consensus sequences constructed from 454 contigs were used to substitute missing BAC sequences. Analyses were not run on genes with sparse or no coverage from 454 sequences.

When polymorphisms were observed between contigs or raw reads within a race or species, several analyses were run on possible variants or haplotypes of the sequence. The preferred method was majority rule, in which the most commonly observed base was selected, but alternative base selections were also tested and the most conservative estimate, or the lowest ω value, was reported. Pairwise and background estimates of ω were plotted across the *HmYb *and *HmB *loci (Figure 2, 3). Gene annotation images of the *HmYb *and *HmB *BAC walks are previously published [17,18].

### Amplifying candidate genes from related species of Heliconiini

Pupae of *H. m. cythera*, *H. m. plesseni, H. numata, Heliconius erato lativitta, Heliconius erato cyrbia, Heliconius himera, Heliconius doris, Heliconius charitonia, Heliconius sara, Heliconius hecale*, and *Dione juno *were purchased from Stratford Butterfly Farm, UK. Forewings, hindwings, and thorax tissue were dissected at the early pupal (EP) and early melanin (EM) stages to be stored in RNAlater at -80 C (for staging see [39]).

Total RNA was extracted from whole forewing and hindwing tissue separately using TRIzol reagent (Invitrogen) and an RNeasy kit (Qiagen), and treated with DNAse using a TURBO DNA-*free *kit (Ambion). RNA samples (2 ug) were reversed transcribed into cDNA using Bioscript (Bioline) and random hexamer primers. Genomic DNA (gDNA) was extracted from <25 mg of thoracic tissue for each species/race using a DNeasy Kit (Qiagen). Final DNA concentration was adjusted to 25 ng/ul for all subsequent RT-PCR reactions. The *RPS13 *housekeeping gene was amplified from all cDNA and gDNA samples as a positive control.

For each of the eight candidate genes that were selected based on preliminary tests of selection or previous population genetics analyses, multiple primers were designed using the BAC sequences to amplify these genes from extrated cDNA and gDNA (Additional file 3). Primers were initially tested with EP and EM cDNA. Polymerase Chain Reactions (PCR) contained 20-50 ng of cDNA, 1x reaction buffer, 2.0 mM MgCl_2_, 0.2 mM dNTPs, 50 pmol of each primer, 0.1 ul of Taq polymerase (Bio-Line) and were run in a thermal cycler with the following conditions: 2 min at 94°C, 40 cycles of 20 s at 94°C, 30 s at 55°C, 2 min at 72°C, and 72°C final extension. PCR products were visualized on a 1.5% agarose gel. When no PCR product bands were observed, PCR experiments were repeated at lower annealing temperatures (51 - 49°C). If amplification continued to prove unsuccessful, likely due to developmental stage specific gene expression, PCR experiments were repeated with gDNA and gDNA-specific primers.

When multiple bands were observed due to non-specific amplification and/or amplification of two alleles with different intronic insertions or deletions, bands were cut from the gel and PCR products extracted using NucleoSpin Extract II Kit (Macherey-Nagel). If multiple PCR products were too similar in size to cleanly separate by cutting, PCR products were cloned using either a TOPO TA cloning kit with one-shot chemically competent cells (Invitrogen) or a pGEM-T easy cloning kit with JM-109 chemically competent cells (Promega). Depending on the number of different PCR products observed in each reaction, 5 to 20 colonies were chosen per transformation, and cells were collected, suspended in 50 ul of sterile water, lysed for 5 minutes at 95°C, and used directly as template in subsequent PCR reactions with either M13 or T7/SP6 primers. PCR products were prepared using a standard big dye protocol before sequencing using an ABI13730 capillary sequencer.

All amplified sequences not previously available were deposited in Genbank. Accession numbers are as follows: ***HM00001 (trehalase 1a) ***[*Heliconius melpomene plesseni *(HQ328814), *Heliconius doris*, (HQ328815), *Heliconius erato lativitta *(HQ328816), *Heliconius sara *(HQ328817), *Heliconius charithonia *(HQ328818)]; ***HM00021 *(*hypothetical protein*) **[*H. doris *(HQ328805), *H. m. plessseni *(HQ328806), *Heliconius erato cyrbia *(HQ328807), *H. e. lativitta *(HQ328808), *Heliconius himera *(HQ328809), *H. charithonia *(HQ328810), *H. sara *(HQ328811)]; ***HM00024 *(*LRR*) **[*H. m. plesseni *(HQ328812), *H. charithonia *(HQ328813)]; ***HM01000 *(*MAX-dimerization*) **[*H. himera *(HQ328819), *H. e. cyrbia *(HQ328820), *H. charithonia *(HQ328821), *H. e. plesseni *(HQ328822), *Dione juno *(HQ328823)]; ***HM01018 *(*rab-kinesin*) **[*H. doris *(HQ328824), *H. charithonia *(HQ328825), *H. e. cyrbia *(HQ328826), *H. e. lativitta *(HQ328827)]; ***HM01022 *(*ashwin*) **[*H. e. plesseni *(HQ328828), *H. doris *(HQ328829), *H. himera *(HQ328830), *H. charithonia *(HQ328831), *H. e. lativitta *(HQ328832), *H. e. cyrbia *(HQ328833)].

### Tests of selection across closely and distantly related species/races

Sequence traces were trimmed and contigs of each species were assembled for each gene using CodonCode Aligner software. Phylogenetic trees were newly constructed for each gene using Maximum Likelihood (ML) in Phylip 3.69 (Joseph Felsenstein, University of Washington). The dnaml program with the JTT (Jones Taylor Thornton) model category was used to calculate distances. Bootstrap re-sampling was done with 100 replicates using the Seqboot program. Matrices were then transformed into trees using the Fitch-Margoliash tree drawing method without molecular clock, and a consensus tree was produced using the Consense program.

The topologies of these genealogies were compared to the most recent published studies of *Heliconius *phylogenetics [26]. When the two topologies differed, the Phylip generated candidate gene tree was used as the tree file for PAML analyses. However, nodes with bootstrap values below 50 were resolved using known phylogenetic relationships between species [26].

When nucleotide sequences contained many indels (for *kinesin *and *HM00021*), protein translations were aligned using ClustalW and non-overlapping regions excluded from analyses (see Additional files 1, 2 for alignments). All available contigs for each gene were formatted into single infiles compatible with PAML.

Tests of selection were run using the CODEML program in PAML, using the *one-ratio *model (NSsites = 0; model = 0) to produce a single *d_N_*/*d_S _*(ω) for each tree, and the *free-ratios *model (NSsites = 0; model = 1), which calculates different ω values for each branch. Analyses were again rerun with model = 2, or the *multiple-ratios *model, producing different ω for groups labelled in the tree file. To compare rates of evolution in the 'diverse lineages', *H. melpomene, H. numata*, and *H. erato*, branches in these clades were assigned separate labels and ω values estimated separately.

The effect of recombination events on detecting positively selected sites for the six selected candidate genes was investigated using GARD (Genetic Algorithms for Recombination Detection) available on the http://datamonkey.org HyPhy webserver [28]. AIC_C _scores rank goodness of fit of a model, with lower scores being better. ΔAIC_C _is a measure of fit of a model accounting for recombination breakpoints, with the greater the difference between the AIC_C _scores of the null and recombinant models, the better the fit. KH tests determine statistical significance of incongruence between tree topologies generated using recombinant fragments and are reported in addition to ΔAIC_C _scores for model validation. GARD topologies generated using recombinant fragments were then used for FEL (Fixed Effects Likelihood) tests of selection [40].

## Authors' contributions

GCW carried out molecular and genetic experiments, participated in the design and coordination of the study, analyzed the data, and drafted the manuscript. CDJ conceived of the study, participated in its design, and helped to draft the manuscript. MJ participated in the design of the study and helped to draft the manuscript. All authors read and approved the final manuscript.

## Supplementary Material

Additional file 1 **ClustalW amino acid alignment of exon 13 of *kinesin *(*HmB*)**. The alignment reveals at least 12 discrete regions of indels (A). All nucleotide sequences, despite the abundance of indels, maintain open reading frames until the end of the exon. In comparison to the remainder of this large gene, the vast majority of indels are found within exon 13, which is the penultimate and largest exon. Though overall sequence alignment predicts greater similarity between *H. erato *and *H. charithonia *(B), an examination of the pattern of indels suggests that *H. melpomene *and *H. charithonia *share a similar pattern. Blue boxes indicate shared sequence deletion between *H. melpomene *and *H. charithonia*, and orange boxes indicate shared insertion of amino acids (A). An unrooted phylogram of exon 13 constructed using the dnaml program in Phylip reveals larger *H. doris *sequence divergence (C). Click here for file

Additional file 2 **ClustalW amino acid alignment of exon 5 of *HM00021 *(*HmYb*)**. Alignment reveals four regions of insertions and deletions (A). All nucleotide sequences, despite the indels, maintain open reading frames until the end of the exon. All indels are found within exon 5, which is the last and largest exon, and the region with the most 454 contig support. A comparison of alignment scores indicates that *H. erato *and *H. melpomene *share highest sequence similarity and that *H. doris *has the most divergent sequence (B). Click here for file

Additional file 3 **Primers used for amplifying candidate genes from related Heliconiini**. Click here for file
