# Supplementary material for: Signatures of selection in loci governing major colour patterns in Heliconius butterflies and related species
Source: BMC Evol Biol. 2010 Nov 29;10:368. doi: 10.1186/1471-2148-10-368 (PMC3001726; doi:10.1186/1471-2148-10-368)

**A**

```
CLUSTAL W (1.83) multiple sequence alignment

kinesin_Herato              NESLEPIFKKEDINRSRMIQQSAINDENSFSSDHSDNSVLNKTTGTLKDENINDSNDNDC 60
kinesin_Hcharithonia        NESLEPIFKKEDINRSRMIQQSAINDENSFSSDHSDNSVLNKTTGXLKDESINNSNGNDC 60
kinesin_Hmelpomene          NESLEPIFKREDINRSRMIHQSAIIDENSLSSDLSDNSVLNKTYGTLKDENINNSNGNDC 60
kinesin_doris               NENLEPIFKKQDINRSKMVQQSAINEKNRLSCDYSNNSLLNKTTGTLKDKNINDSNCIDC 60
                            **.******::*****:*::****  ::*  :*.*  *:**:**** * ***:.**:**   **

kinesin_Herato              TFETGN-DVSANNKSFNDNKEPEVVTTEVNIDVDNDIEFKKDIQLSRDTYFVENNDHIVD 119
kinesin_Hcharithonia        TFETGX-DVSAN----------EVITTEVNIDVNNDIEFEKDIKLSRDTYFVENNDCFVN 109
kinesin_Hmelpomene          TFETDN-DVVAG----------EVVTTELNIDVHNNIEFKNHIKLSRETYLVENNDSFVN 109
kinesin_doris               ENNNDGDDFSANNESFNDNNEPEVVTTELNIDVHKDKEFKEDIKLSRETYFVENNYSFVN 120
                              :..   *.  *.          **:***:****.::  **::.*:***:**:****   :*:

kinesin_Herato              DKKIVIS--LSRATYCVSSNDDNVLDMDNKSQSNRNSLKYIMAKDSALETDNNNKPNTFI 177
kinesin_Hcharithonia        DKKKVL---LSR--LLSANNDDNVIDIDNKPKSNKN---RNSLKDSPLETDNNNKPNTFI 161
kinesin_Hmelpomene          DKKKVLVSTLSR-----ASNEDIVVDINDKTKTNKN---RNSFKDSSLETVHNNKPNTFV 161
kinesin_doris               NNKKVSS---VRGTYSFRNNEDN-VDIYNKSISNRNFLKDNVAKDSSLGTDDNNKPNTFI 176
                            ::* *        *         .*:*   :*:  :*.  :*:*     ***.* *  .*******:

kinesin_Herato              NNIISSIKESNLNTQSNNSLTLFEKLEEATKNDTDTNSNATSFFNIKKLKEKRNFFGRDD 237
kinesin_Hcharithonia        NNIISCIKESNLNTQSNNSLTLFEKLEEATKTDTDTNSNDTSFFNIKKLKEKRNFFGRDD 221
kinesin_Hmelpomene          NNIINSIKESNLSIQSNNSLASFEKLEEATKIDTGTNSNATNFFNIKILKEKRNFFNYDD 221
kinesin_doris               NNIISSIKESNLSIQSNNSLALFEKLEEVTKTDTDTNSMDTSFFNIKILKEKRNLFDHGN 236
                            ****..******. ******: ******.** **.***  *.***** ******:*.  .:

kinesin_Herato              VPKSPETPATVVKCKDRKIYFDN-IDNSS-NNDEDKVKIDDYRSPSIVKDDVTNDFEPSM 295
kinesin_Hcharithonia        VPKSPETPATVVKCKDRKIYFDNSIDNSSSNNDNEKVKIDDYRSPSIVKDDVTNDFEPSM 281
kinesin_Hmelpomene          VLKSLEKPATIVKCKDKKIYFDNSNNNDISNNDNEKIKIDDYRSPSIVKEDVTNDYEPSM 281
kinesin_doris               VTKSPKTPTTVVKCKDKKIYFDN--VDTSSSNDNDKVEINDYRSPSIVKDDVTNDFEPSM 294
                            * **  :.*:*:*****:******     :    .**::*::*:*********:*****:****

kinesin_Herato              IKKLLGQSITNNADNLN--AFQKIKICDNKNDSIDLFEALDSPQACNK--VQDDIENVRK 351
kinesin_Hcharithonia        IKKLLGQSVTNQPDNLN--ALQKNKICEKKNDSIDLFEALDSPQACNKNTVNDDIENVRK 339
kinesin_Hmelpomene          IKKLLGQSITNQNDNLN--ALQKIRTCDKKNDSIDLFEALDSPQACNKNKVNDDIENIRK 339
kinesin_doris               IKKLLG-DITNQVDNTKSLNLQKGRTCDKTHDSIYLFEGLYLPQVCNK--VKDDTENVRK 351
                            ****** .:**: **  :    :** : *::.:*** ***.*   **.***   *:** **:**

kinesin_Herato              SIQNVAILPEDGIACKNIDAEHNQKIVSNDELNDVEETNDNERVEEIVNNIDVESNESNS 411
kinesin_Hcharithonia        SIQNVAIIQEDAIVCKNI--EHNQXIVSNDGLNDVEEXNGNKIVEEIVNNIDVDSNKGNS 397
kinesin_Hmelpomene          SVQNVAIIQEDAIVCGNI--EHNQEIVSNDELNNVEETNENKHVKEIVNDVDVESNKDNT 397
kinesin_doris               YIQNVGIKQEDTIVCENIDIEQNQEIISNDELNNEEETNENKHVK-LFNDVNVGNNKDIS 410
                             :***.*   ** *.* **   *:** *:*** **: ** * *: *: :.*:::* .*:. :

kinesin_Herato              QNKEIISKISIENTDEIEKVNNDESQLQINNTKTDNTLDEFENIYKDITTSRATEFDLLV 471
kinesin_Hcharithonia        QNNEIITKKSIQNSDEIKKVNYDESQLQINNTNTDNTLDAFENIYKDITTSRATEFDLLV 457
kinesin_Hmelpomene          IKKEETMKISLERSDDIEKVSNDETQLLINNTKSDNTSDEFENIYKDITTPRATEFDLLV 457
kinesin_doris               ENKEITMQMCIENSDEIEKLNNYESQLNMNNTKIDNTLDEFANIYKDMTTSRATEFDLLL 470
                             ::*   : .::.:*:*:*:.  *:** :***: *** * * *****:**.********:

kinesin_Herato              SQEIKDCTIDTTNTNNETENEDLKYNLRQKSKMDKPKNQTRRGKKVLECSPPKSETAKPK 531
kinesin_Hcharithonia        SQEIKDSTIDTT-TNNETENEDLKYNLRQKSKMDKPKNHTRRGKKVSESSPPKSEKAKPN 516
kinesin_Hmelpomene          SQEIKDTTIDTT-TNNEKENEDLKYNLRQKSKMDKPKNETRRGKKVLESSPPKCEKSKTR 516
kinesin_doris               SQEIKDSTIDPNNITDETENEDLKYNLRQKSKIIKPKNETRRGKKVSESSPPKDEKTKLK 530
                            ****** ***..  .:*.**************: ****.******* *.**** *.:* .

kinesin_Herato              KRNLRLRRRQNASEDNSEDGSKDRSKDESKDIDADTNVKDIEKAKLKDIINLQKEFSDVT 591
kinesin_Hcharithonia        KRNLRLRRQ---SEDNSEDGSKD---------IDADTKEKDIEKAKXXDIRXXQNEFSDVT 565
kinesin_Hmelpomene          KRNLRLRRQ---CGDNSEDGSKD---------IDEDTKKRDIEKAKLKDIINLQKEFSDVT 565
kinesin_doris               KRNLRLRRRRNLSEDSSEDGSKD----ESKDIDTNTKEKDIEITKSRYIINLQKEFSDVT 586
                            ********:   . *.*******         ** :*: :*** :*   *   *:******

kinesin_Herato              LDKPAPAKEFKDIPS 606
kinesin_Hcharithonia        LDKXXXXKEFKDIPS 580
kinesin_Hmelpomene          LDKPAPAKEFKDIPS 580
kinesin_doris               LDKPAPATEFKD--- 598
                            ***    .****
```

**B**

| SeqA | Name | Len(aa) | SeqB | Name | Len(aa) | Score |
|---|---|---|---|---|---|---|
| 1 | kinesin_Herato | 606 | 2 | kinesin_Hcharithonia | 580 | 83 |
| 1 | kinesin_Herato | 606 | 3 | kinesin_Hmelpomene | 580 | 76 |
| 1 | kinesin_Herato | 606 | 4 | kinesin_doris | 598 | 70 |
| 2 | kinesin_Hcharithonia | 580 | 3 | kinesin_Hmelpomene | 580 | 77 |
| 2 | kinesin_Hcharithonia | 580 | 4 | kinesin_doris | 598 | 66 |
| 3 | kinesin_Hmelpomene | 580 | 4 | kinesin_doris | 598 | 67 |

**C**

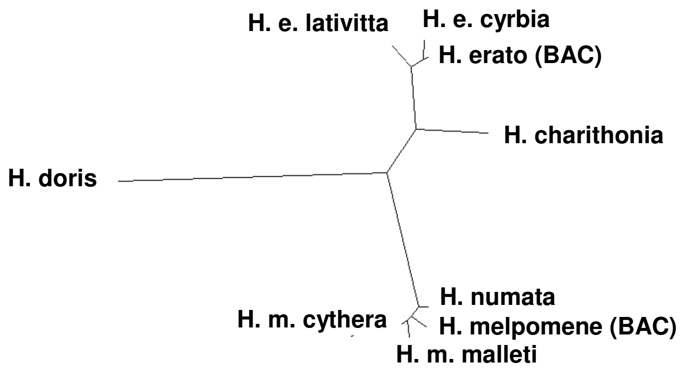

Supplement: Additional file 1 — ClustalW amino acid alignment of exon 13 of kinesin (HmB). The alignment reveals at least 12 discrete regions of indels (A). All nucleotide sequences, despite the abundance of indels, maintain open reading frames until the end of the exon. In comparison to the remainder of this large gene, the vast majority of indels are found within exon 13, which is the penultimate and largest exon. Though overall sequence alignment predicts greater similarity between H. erato and H. charithonia (B), an examination of the pattern of indels suggests that H. melpomene and H. charithonia share a similar pattern. Blue boxes indicate shared sequence deletion between H. melpomene and H. charithonia, and orange boxes indicate shared insertion of amino acids (A). An unrooted phylogram of exon 13 constructed using the dnaml program in Phylip reveals larger H. doris sequence divergence (C). [file 1471-2148-10-368-S1.PDF]
