# Supplementary material for: Signatures of selection in loci governing major colour patterns in Heliconius butterflies and related species
Source: BMC Evol Biol. 2010 Nov 29;10:368. doi: 10.1186/1471-2148-10-368 (PMC3001726; doi:10.1186/1471-2148-10-368)
Supplement: Additional file 2 — ClustalW amino acid alignment of exon 5 of HM00021 (HmYb). Alignment reveals four regions of insertions and deletions (A). All nucleotide sequences, despite the indels, maintain open reading frames until the end of the exon. All indels are found within exon 5, which is the last and largest exon, and the region with the most 454 contig support. A comparison of alignment scores indicates that H. erato and H. melpomene share highest sequence similarity and that H. doris has the most divergent sequence (B). [file 1471-2148-10-368-S2.PDF]

A

```
HM00021_Hera          IHKPQDIDTNEVSSPKKSRKNKDASGTKVKRSKRKIDDNIIIENDCENQPIEENNDIIQR 60
HM00021_charitonia    IDKSRNIDINEVSSPKKSRKNKDASGKKVKRSKIKIDGNIITDNDCENQPXEVNNDIIQX 60
HM0001_Hmel           IHKPPDIDTKEVSSPKKSRKNKDASGKKVKRSKRKIDDNIITDNDCENQPTEENNDIIQK 60
HM00021_doris         VGLLTDIGTNEVSSPKKSKKNKDASGRKVKRTKRKIDDNIITDNDCKTQPIEENNDVIQK 60
                      :    :*. :********:******* ****:* ***.*** :***:.** * ***:**

HM00021_Hera          NNDHIEITAGQNKHNENKNSESILENYENVSDINMNQDDLTEEFTFGNAAQDVVENENIL 120
HM00021_charitonia    NNDHIDISADXNKHNENKNSESILGNYENVSDINMDQDGLTEEFTFGNAAQDIVENENIL 120
HM0001_Hmel           NNDHIDITADQNKHNENENSESTLENYENISDINMNQDNLTEEFTFGNAAQDIVEMKNIL 120
HM00021_doris         NDDHIEITSDQNKDNENENSESILETYGNVSDINMYQDGLTEEFTFGNAAQDIVETENIL 120
                      *:***:*::. **.***:**** * .* *:***** **.*************:** :***

HM00021_Hera          SNKSTESIDFDEFAIKINNND----VNEKDCKENSEECDNRND--KCQREEKNTDNVECF 174
HM00021_charitonia    SNKSTESIDFDEIEKKINNNDAYDQINEKDYKKNSVECVKSDD--KCQREKKNTDNDDCL 178
HM0001_Hmel           SNKSTESIDFDEIAMKIDNND----VNDKDCKDTLEECNESND--KCQREEKLTDNDEYF 174
HM00021_doris         SNKSTESIDFDEIEMKINNNDICDEIDDKDYEDTSKECHESHERNKCQKGEKNT---ECF 177
                      ************:  **:***    :::** :..  ** : .:  ***: :* *   : :

HM00021_Hera          VKCDT-KSADSKLTDVVDIGELGNTAVKNNLDLKENIDVKETGTELEINDECHKDIDAKI 233
HM00021_charitonia    QKCD------SKLTDIVDTGELGNRAVKKHLVLKENIDVKETGTELEINGECHKDIDAKI 232
HM0001_Hmel           KMCDT-KSADSKPTDSMDIGKLGDSVVKNDLVLKESIDIKQTGTELDINGECHKDIDAKI 233
HM00021_doris         VKCDKSKSADSKPTNSMDIGELGKTVVKNDLVLKESIDLKETGTELEMNGKCHKDIDA-- 235
                        **      ** *: :* *:**. .**:.* ***.**:*:*****::*.:*******
```

B

SeqA	Name	Len(aa)	SeqB	Name	Len(aa)	Score
1	HM00021_doris	235	2	HM00021_Hera	242	70
1	HM00021_doris	235	3	HM00021_charitonia	241	67
1	HM00021_doris	235	4	HM0001_Hmel	242	70
2	HM00021_Hera	242	3	HM00021_charitonia	241	80
2	HM00021_Hera	242	4	HM0001_Hmel	242	81
3	HM00021_charitonia	241	4	HM0001_Hmel	242	75
